# SNP-mediated lncRNA-ENTPD3-AS1 upregulation suppresses renal cell carcinoma via miR-155/HIF-1α signaling

**DOI:** 10.1038/s41419-021-03958-4

**Published:** 2021-07-03

**Authors:** Jiangyi Wang, Yun Zou, Bowen Du, Wenzhi Li, Guopeng Yu, Long Li, Lin Zhou, Xin Gu, Shangqing Song, Yushan Liu, Wenquan Zhou, Bin Xu, Zhong Wang

**Affiliations:** 1 Department of Urology, Shanghai Ninth People’s Hospital, Shanghai Jiaotong University School of Medicine, Shanghai, 200011 China; 2 Department of Urology, Jinling Hospital, Medical School, Nanjing University, Nanjing, China

**Keywords:** Cancer epigenetics, Renal cell carcinoma

## Abstract

Over the last decade, more than 10 independent SNPs have been discovered to be associated with the risk of renal cell carcinoma among different populations. However, the biological functions of them remain poorly understood. In this study, we performed eQTL analysis, ChIP-PCR, luciferase reporter assay, and Cox regression analysis to identify the functional role and underlying mechanism of rs67311347 in RCC. The ENCORI database, which contains the lncRNA–miRNA–mRNA interactions, was used to explore the possible target miRNA of ENTPD3-AS1. The results showed that the G > A mutation of rs67311347 created a binding motif of ZNF8 and subsequently upregulated ENTPD3-AS1 expression by acting as an enhancer. The TCGA-KIRC and our cohorts both confirmed the downregulation of ENTPD3-AS1 in RCC tissues and demonstrated that increased ENTPD3-AS1 expression was associated with good OS and PFS. Furthermore, ENTPD3-AS1 interacted with miR-155-5p and activated the expression of HIF-1α, which was an important tumor suppressor gene in the development of RCC. The functional experiments revealed that overexpression of ENTPD3-AS1 inhibited cell proliferation in RCC cell lines and the effect could be rescued by knocking down HIF-1α. Our findings reveal that SNP-mediated lncRNA-ENTPD3-AS1 upregulation suppresses renal cell carcinoma via miR-155/HIF-1α signaling.

## Introduction

Renal cell carcinoma (RCC) is the most lethal cancer in the genitourinary system, with 73,750 new cases diagnosed and 14,830 deaths confirmed in the United States in 2020 [1]. Up to 16% of patients diagnosed with RCC are found distantly metastatic, and the 5-year survival rate is only 13% [1]. Although immunotherapy combined with targeted therapy has improved the prognosis of metastatic patients, the overall survival is only 48 months [2–6]. Furthermore, drug resistance and immune-related adverse events (irAEs) are important problems in clinical practice. Thus, exploring the pathogenesis of RCC and new therapeutic strategies remains an issue of great challenge.

Several factors have been reported to contribute to the initiation and progression of RCC, among which the genetic heritability likely played a significant role [7]. Although more than ten germline alterations (VHL, MET, FLCN, FH, etc) are associated with a strong risk of RCC, they collectively cannot account for the twofold increased risk of RCC seen in first-degree relatives of RCC patients [8, 9]. There are likely other genetic components or polymorphisms that are heritable and may confer a mildly increased risk [10]. Over the last decade, genome-wide association studies (GWAS) have identified thousands of SNPs associated with predisposition to disease [11]. More than 90% of these SNPs are located in noncoding regions [12, 13]. As to RCC, over 10 independent SNPs have been discovered to be associated with the disease among different populations [14–18]. Recently, a meta-analysis including 10,784 cases and 20,406 controls of European ancestry confirmed rs67311347 as an RCC risk loci [19]. However, the biological functions of this RCC-related SNP remain poorly understood.

During the past decade, evidence has accumulated showing a crucial role for several lncRNAs in the pathogenesis of RCC, including H19, MALAT1, HOTAIR, and lncRNA-SARCC. These lncRNAs participate in the biological process of RCC through HIF-related and HIF-independent pathways. Furthermore, risk-related SNP rs11672691 can remotely modulate the expression of lncRNA PCAT19, then promoting the progression of aggressive prostate cancer [20]. Several studies have also revealed that the disease-associated SNPs might function as a transcriptional enhancer, and interact with transcriptional factors to modulate the expression of target genes [21, 22]. Thus, we hypothesize that RCC-related SNP rs67311347 might also contribute to RCC initiation by regulating the expression of lncRNAs through a remote modulation.

In the current study, we first identify that rs67311347 contributes to the pathogenesis of RCC by promoting the expression of lncRNA-ENTPD3-AS1 through functioning as an enhancer. Next, we found that low expression of ENPTD3-AS1 was associated with poor survival of patients with RCC. Further experiments revealed that ENTPD3-AS1 inhibited RCC development by upregulating the expression of HIF1A by interacting with miR-155-5p. The results will fill the gap between RCC-related SNP and the disease-associated phenotype and will be important in understanding the pathogenesis of RCC and facilitating new strategies for prevention and treatment.

## Results

### SNP rs67311347 regulated the expression of lncRNA ENTPD3-AS1 as an enhancer

**Fig. 1:**
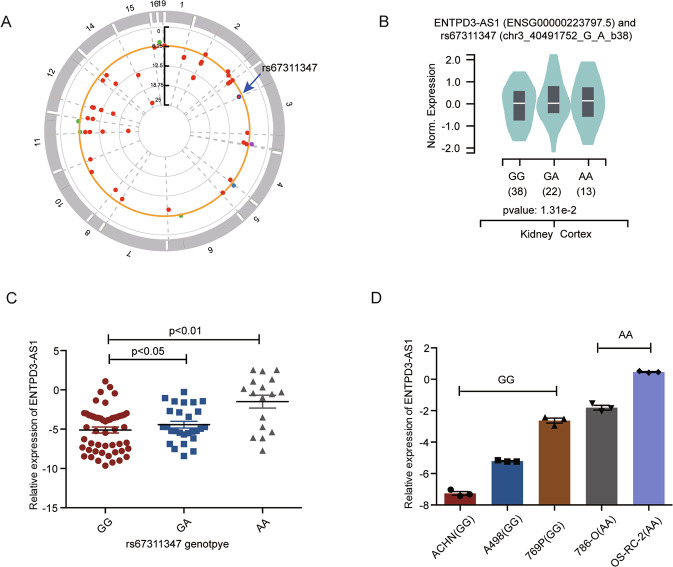
RCC-associated SNP rs67311347 regulated the expression of ENPTD3-AS1. **A** The Manhattan Plot of RCC-associated SNPs in GWAS. **B** eQTL analysis of rs67311347 using data from the GTEx Project. **C** The expression of ENPTD3-AS1 in normal kidney tissues with different genotypes of SNP rs67311347 in Cohort 1. **D** The expression of ENPTD3-AS1 in RCC cell lines with different genotypes of SNP rs67311347.

SNP rs67311347 had been reported to be related to the risk of renal cell carcinoma in a large meta-analysis of GWAS (OR = 0.90, *p* = 2.5 × 10^−8^) (Fig. 1A). However, the underlying mechanism remains unclear. The expression quantitative trait loci (eQTL) analysis could provide valuable information for the biological basis for disease association identified through GWAS studies [23]. cis-eQTL revealed the most significant association between rs67311347 and ENTPD3-AS1 in multiple normal tissues including kidney-cortex according to the Genotype-Tissue Expression (GTEx) project (Fig. 1B and Fig. S1). In line with the GTEx data, kidney samples with genotype AA or GA had higher expression of ENTPD3-AS1 compared to those with genotype GG (Fig. 1C). To further confirm this association, we examined the genotype of common RCC cell lines (786-O, OS-RC-2, 769 P, A498, and ACHN) and found that RCC cells with genotype AA (786-O and OS-RC-2) showed higher expression of ENTPD3-AS1 than those with genotype GG (769 P, A498, and ACHN) (Fig. 1D). Taken together, rs67311347 might lead to RCC by regulating the expression of ENTPD3-AS1.

**Fig. 2:**
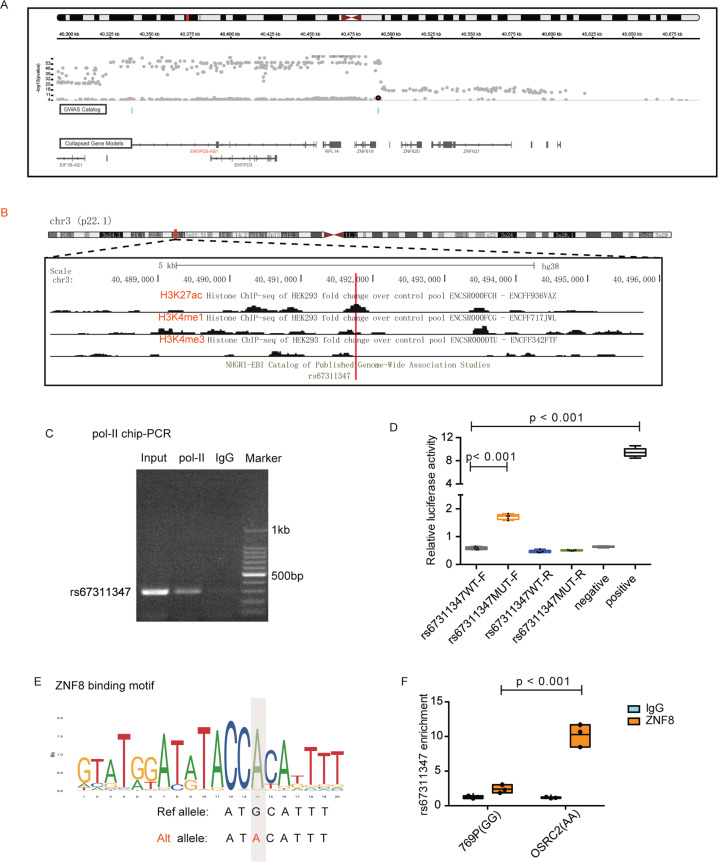
SNP rs67311347 regulated the expression of lncRNA ENTPD3-AS1 as an enhancer. **A** The genomic location of rs67311347 and ENTPD3-AS1. **B** Epigenetic tracks obtained from ENCODE database showed the enrichment of enhancer marks (H3K27ac peaks, H3K4me1 peaks) and transcription marks (H3K4me3 peaks) in the rs67311347 region. **C** The Polymerase II ChIP-PCR showed that Polymerase II occupied the rs67311347 region. **D** Relative reporter gene activity of the constructs containing the WT or Mut allele of rs67311347 in forward orientation in A498 cells. **E** The altered allele A of rs67311347 created a ZNF8 binding motif. **F** ChIP-qPCR of ZNF8 in RCC cell lines carrying different genotypes of rs67311347.

Functional SNPs are believed to modify the activity of transcriptional regulatory regions [24]. Considering that rs67311347 was located in 3p22.1, which was about 30 kb away from the 5′ end of ENTPD3-AS1 (Fig. 2A), we assumed that rs67311347 might function as an enhancer and regulate ENTPD3-AS1 expression. To elucidate the hypothesis, we downloaded the H3K27ac, H3K4me1, and H3K4me3 ChIP-seq data from the ENCODE project. The results showed that the rs67311347 region was enriched with H3K27ac and H3K4me1 markers, while not enriched with H3K4me3 (Fig. 2B). We further performed the Polymerase II ChIP-PCR, and the result showed that Polymerase II occupied on the rs67311347 region (Fig. 2C). Then, we performed luciferase-based enhancer assays by cloning the rs67311347 region (wild-type and mutated) into the reporter vectors. The forward mutated region had significantly higher activity than the wild type one (Fig. 2D). All the above results suggested that rs67311347 regulated ENTPD3-AS1 expression as an enhancer.

### SNP rs67311347 modified the binding site of ZNF8

In the previous study, SNP-specific changes were reported to regulate enhancer activity by altering transcription factor binding [25]. Thus, we examined whether rs67311347 directly modified the DNA-binding motif through a reliable method called FIMO. The result indicated that rs67311347 and its flanking sequences overlapped with the binding motif of transcriptional factor ZNF8 (Fig. 2E). In detail, ZNF8 had a higher preference for the altered allele “A”. Consistent with the FIMO analysis, ChIP-PCR also showed that a stronger ZNF8 binding was enriched in the rs67311347 region in OSRC-2 cells with AA alleles than in 769 P cells with genotype GG (Fig. 2F). Furthermore, the expression of ENTPD3-AS1 was positively associated with ZNF8 (*p* < 0.01, Fig. S2A), and higher expression of ZNF8 was related to longer PFS in the TCGA-KIRC cohort (Fig. S2B). Altogether, we demonstrated that the SNP rs67311347 functioned as an enhancer and promoted the expression of ENTPD3-AS1 through modifying ZNF8 binding.

### ENTPD3-AS1 was a protective lncRNA in RCC development

**Fig. 3:**
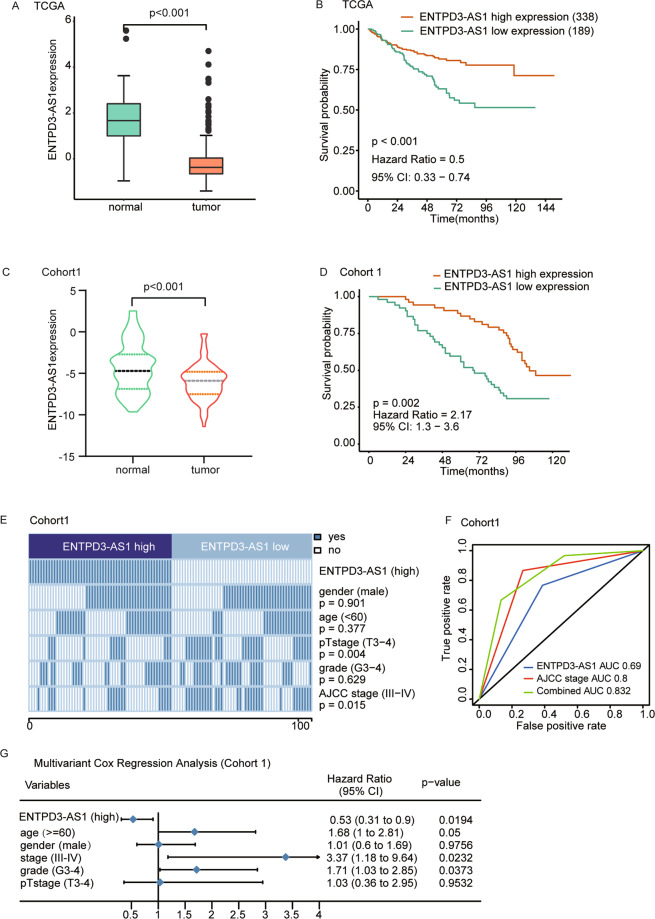
ENTPD3-AS1 was a protective lncRNA in RCC development. **A** ENTPD3-AS1 expression in the tumor and normal tissues in TCGA-KIRC. **B** The Kaplan–Meier analyses of overall survival between patients with high and low expression of ENTPD3-AS1 in TCGA-KIRC, *n* = 527. **C** ENTPD3-AS1 expression in RCC tissues and adjacent normal tissues in Cohort 1. **D** The Kaplan–Meier analyses of overall survival between patients with high and low expression of ENTPD3-AS1 in Cohort 1, *n* = 105. **E** Clinical and pathological features between ENTPD3-AS1 high and low-expression groups in Cohort 1. The heatmap demonstrated the association of different characters with ENTPD3-AS1 expression. The *χ*2 test was used. **F** The AUC of overall survival for ENTPD3-AS1 expression, AJCC stage, and combined group in Cohort 1. **G** Multivariate cox regression analysis of ENTPD3-AS1 expression in Cohort 1.

**Fig. 4:**
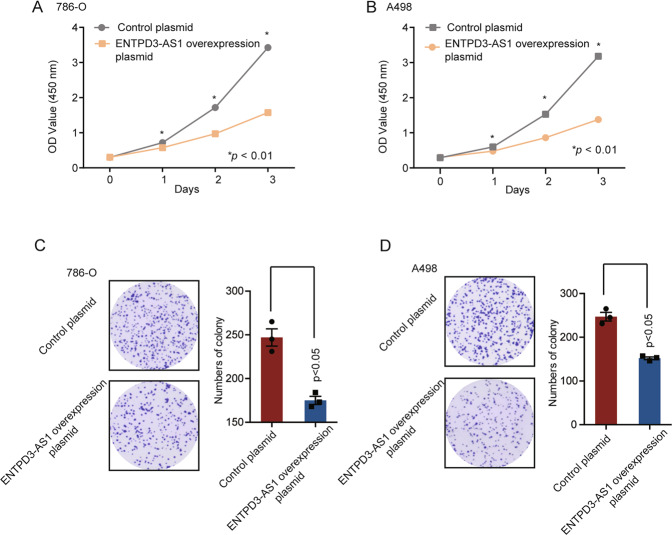
The in vitro functional assays of ENTPD3-AS1. **A**, **B** Cell proliferation was measured in 786-O and A498 cells after being transfected with ENTPD3-AS1 overexpression plasmid. **C**, **D** Colony formation was measured in 786-O and A498 cells after being transfected with ENTPD3-AS1 overexpression plasmid.

Next, we evaluated the role of ENTPD3-AS1, a long non-coding RNA, in the initiation and progression of RCC. The results showed that ENTPD3-AS1 expression was significantly lower in RCC tissues compared to that in the adjacent normal tissues in the TCGA-KIRC cohort (Fig. 3A), and lower expression of ENTPD3-AS1 was related to pT3–pT4 patients (Fig. S3A). The Kaplan–Meier analyses and univariant Cox regression analyses showed that high expression of ENTPD3-AS1 was associated with a favorable prognosis in RCC patients (HR = 0.5, *p* < 0.001) (Fig. 3B, Fig. S3B). To further confirm the results, the clinical and expression data from cohort 1 were analyzed. As expected, ENTPD3-AS1 expression was decreased in RCC tissues compared to corresponding normal tissues, and high ENTPD3-AS1 expression predicted a good prognosis (Fig. 3C, D). Besides, the clinicopathological analysis in cohort 1 demonstrated that ENTPD3-AS1 expression was negatively correlated to pT stage and AJCC stage (Fig. 3F), and the AUC of ENTPD3-AS1 expression combined with AJCC stage reached 0.832 in the ROC in cohort 1 (Fig. 3E). Furthermore, the multivariate Cox regression analyses revealed that ENTPD3-AS1 expression was an independent predictor of RCC survival in cohort 1 (HR = 0.53, *p* = 0.0194) (Fig. 3G). The in vitro functional assays validated that overexpression of ENTPD3-AS1 significantly suppressed RCC cell proliferation (Fig. 4A, B) and colony formation (Fig. 4C, D) in 786-O and A498 cells. Meanwhile, knockdown of ENTPD3-AS1 significantly promoted RCC cell proliferation (Fig. S3C, D). Collectively, ENTPD3-AS1 was a lncRNA with tumor suppressor function in RCC development.

### ENTPD3-AS1 suppressed RCC development through a miR-155-5p/HIF-1α pathway

**Fig. 5:**
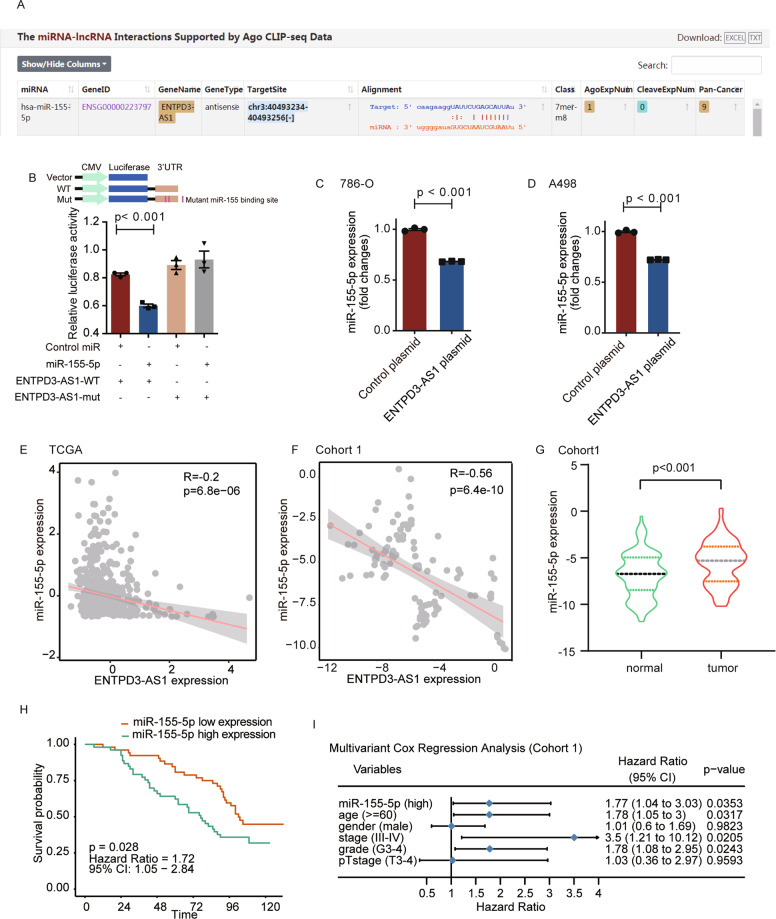
ENTPD3-AS1 suppressed RCC development through miR-155-5p. **A** The predicted target of ENTPD3-AS1 in the ENCORI database. **B** The direct interaction between ENTPD3-AS1 and miR-155-5p was confirmed by luciferase reporter assay. **C**, **D** Overexpression of ENTPD3-AS1 decreased miR-155-5p expression in 786-O and A498 cells. **E** The correlation between ENTPD3-AS1 and miR-155-5p expression in TCGA-KIRC. **F** The correlation between ENTPD3-AS1 and miR-155-5p expression in Cohort 1. **G** The expression of miR-155-5p in RCC tissues and corresponding normal tissues in Cohort1. **H** The Kaplan–Meier analyses of overall survival between patients with high and low expression of miR-155-5p in Cohort 1. **I** Multivariate cox regression analysis of miR-155-5p expression in Cohort 1.

We next explored the potential mechanism by which ENTPD3-AS1 resulted in RCC development. LncRNAs may function as “microRNA sponges” to compete and degrade their targeted microRNAs at the post-transcriptional level. To determine which microRNA interacted with ENTPD3-AS1, we searched for interaction targets of ENTPD3-AS1 in the ENCORI database (http://starbase.sysu.edu.cn/index.php) (Fig. 5A). We found miR-155-5p, which contained the interaction targets with ENTPD3-AS1. To demonstrate the direct interaction between ENTPD3-AS1 and miR-155-5p, we constructed a luciferase reporter plasmid encoding the binding site of ENTPD3-AS1 in the 3′ UTR of the luciferase gene. Luciferase assays showed that miR-155-5p significantly reduced the luciferase activity in comparison to the control miRNA, and the mutation of ENTPD3-AS1 target sequence eliminated the interaction (Fig. 5B). Real-time PCR data further revealed that overexpression of ENTPD3-AS1 decreased miR-155-5p expression in 786-O and A498 cells (Fig. 5C, D).

To better understand the association between ENTPD3-AS1 and miR-155-5p, the expression association of them was analyzed in the TCGA-KIRC cohort and Cohort 1. The results showed that ENTPD3-AS1 levels were negatively correlated with miR-155-5p in RCC tissues (Fig. 5E, F). Besides, miR-155-5p expression was significantly higher in RCC tissues compared with corresponding normal tissues (Fig. 5G). The expression of miR-155-5p was correlated with pathological features and overall survival of TCGA-KIRC (Fig. S5). Meanwhile, we assessed the impact of miR-155-5p on the outcome of patients in Cohort 1. The overall survival was poorer in patients with higher levels of miR-155-5p (Fig. 5H). The multivariate Cox regression analysis further indicated that high miR-155-5p expression was an independent risk factor for survival in RCC patients (Fig. 5I).

**Fig. 6:**
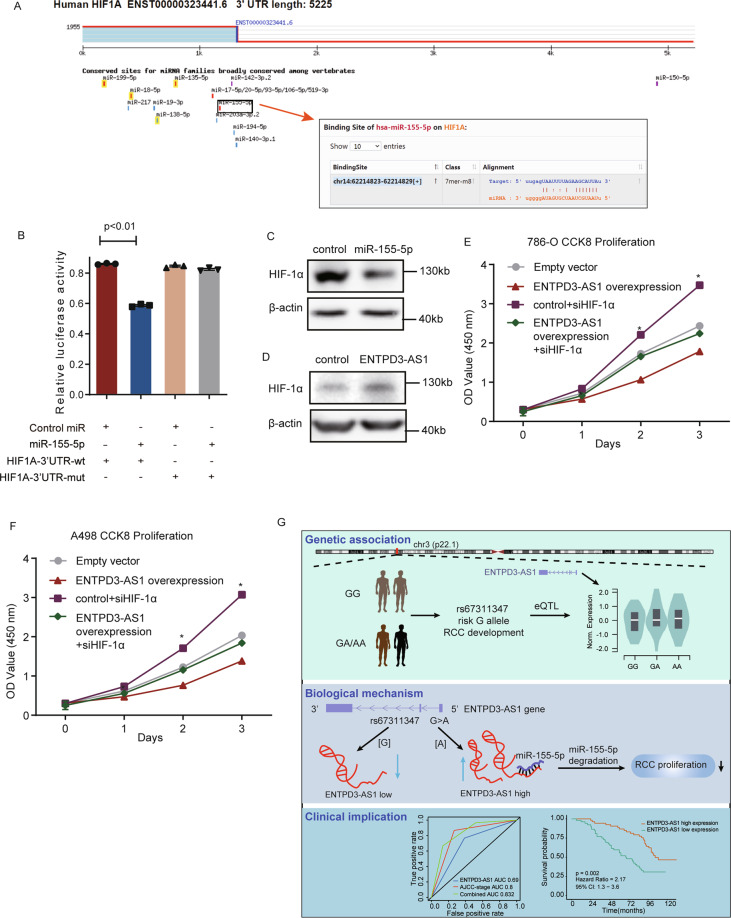
miR-155-5p promoted RCC development by regulating HIF-1α. **A** The potential target gene of miR-155-5p in TargetScanHuman database. **B** The direct interaction between miR-155-5p and HIF-1α by luciferase assays. **C** The expression of HIF-1α after overexpression of miR-155-5p. **D** The expression of HIF-1α after overexpression of ENTPD3-AS1. **E**, **F** Knockdown of HIF-1α rescued ENTPD3-AS1 overexpression inducing a decrease in cell proliferation. **G** SNP-mediated lncRNA-ENTPD3-AS1 upregulation suppresses renal cell carcinoma via miR-155/HIF-1α signaling.

To specifically define the target gene of miR-155-5p, we assessed the TargetScanHuman database (http://www.targetscan.org/vert_72/) and found HIF-1α as the potential target gene of miR-155-5p (Fig. 6A). To demonstrate the direct interaction between miR-155-5p and HIF-1α, we performed luciferase assays. The results showed that miR-155-5p significantly reduced the luciferase activity in comparison to the control miRNA and the mutation of the miR-155-5p target sequence eliminated the interaction (Fig. 6B). Moreover, transfection with miR-155-5p decreased HIF-1α expression, and overexpression of ENTPD3-AS1 increased HIF-1αexpression (Fig. 6C, D). Furthermore, rescue analysis revealed that overexpression of miR-155-5p abolished the increased HIF-1α expression by lncRNA ENTPD3-AS1 (Fig. S6C) and HIF-1α expression was positively associated with the expression of ENTPD3-AS1 in the TCGA-KIRC cohort (Fig. S6D). We next examined whether HIF-1α mediated the biological function of ENTPD3-AS1 in RCC. Knockdown of HIF-1α significantly rescued ENTPD3-AS1 overexpression inducing a decrease in cell proliferation (Fig. 6E, F). Collectively, these data indicated that ENTPD3-AS1 can competitively interact with miR-155-5p and positively regulate the expression of miR-155-5p-targeted gene HIF-1α (Fig. 6G).

## Discussion

Thousands of disease-related SNPs have been identified by GWAS in the last decades, most of which are located in noncoding regions [11]. However, the biological function of the trait-associated SNPs remains unclear. In the previous meta-analysis, rs67311347 at 3p22.1 was confirmed to be associated with the risk of RCC (OR = 0.90, *p* = 2.5 × 10^−8^). In this study, we demonstrated the molecular mechanism and clinical implication of rs67311347 in the development of RCC, which functioned as an enhancer element and regulated the expression of lncRNA ENTPD3-AS1 through the miR-155-5p/HIF-1α pathway.

ENTPD3-AS1 is a long non-coding RNA located at 3p. As is known, the short arm of chromosome 3 is the most important region contributing to the pathogenesis of RCC, for the reason that several tumor suppressor genes, like VHL, SETD2, and PBRM1, are identified within this region [26]. ENTPD3-AS1 was reported to be significantly associated with prostate cancer [27]. A locus (rs193921050) in ENTPD3-AS1 has been reported for “malignant tumor of the prostate” in ClinVar with uncertain clinical significance [28]. However, the role of ENTPD3-AS1 in the development of RCC was unclear. In our study, lower expression of ENTPD3-AS1 was detected in RCC tissues compared to that in corresponding normal tissues and was associated with a poor prognosis, suggesting its tumor suppressor role in RCC. As the loss of heterogeneity of 3p is a common phenomenon in RCC, especially clear cell RCC [29, 30], we speculated that loss of ENTPD3-AS1 might promote tumor development in combination with VHL and other tumor suppressor genes.

LncRNAs played an integral role in a series of pathological processes in tumorigenesis of various human cancers [31–33]. Further studies suggested that lncRNAs could serve as natural miRNA sponges to restrain the function of intracellular miRNA via sharing one or more miRNA response elements (MREs), which was known as the competing endogenous RNA (ceRNA) hypothesis [34]. In this study, we confirmed that ENTPD3-AS1 could directly interact with miR-155-5p, resulting in the upregulation of HIF-1α. Although overexpression of HIF-1a and/or HIF-2a is associated with poor survival in many other types of cancer including colorectal, breast, and prostate cancers, HIF1α has been identified as a suppressor gene for kidney cancer located in 14q [35–37]. Thus, the protective SNP rs67311347 suppresses the occurrence of RCC through an ENTPD3-AS1/miR-155-5p/HIF-1a axis.

The function of disease-associated SNPs differs in their location on the genome. Though in the majority of cases, SNPs regulate the most proximal gene, several variants can mediate distal regulatory effects through long-range chromatin interactions [38]. Some SNPs located in non-coding regions within potential regulatory elements have been reported to distally regulate the expression of target genes and contribute to the pathogenesis of multiple diseases [38]. The regulatory elements could be either promoters or enhancers, or even function as both a promoter and an enhancer [20]. In our study, the A allele of rs67311347 altered the enhancer of lncRNA ENTPD3-AS1 and increased the binding of ZNF8, which was a transcription factor, consequently upregulating the expression of ENTPD3-AS1. In line with our results, Gao et al. demonstrated that rs11672691 resided in an enhancer element and altered the binding site of HOXA2, a novel oncogenic transcription factor with prognostic potential in prostate cancer [39]. Thus, modification of regulatory elements might be a common mechanism of gene expression referring to intergenic SNPs.

In conclusion, we for the first time find that rs67311347 in 3p22.1 upregulates ENTPD3-AS1 expression by acting as a transcriptional enhancer and directly changing the binding efficiency of transcription factor ZNF8. The upregulation of LncRNA ENTPD3-AS1 inhibits cell proliferation and migration through the miR-155-5p/HIF-1α pathway in renal cell carcinoma.

## Materials and methods

### Participants

A total of 105 RCC patients were involved for analysis who underwent surgery between 2009 and 2012 (Cohort 1) from the Shanghai Ninth People’s Hospital, School of Medicine, Shanghai Jiaotong University. The study protocol was approved by the ethics committee of Shanghai Ninth People’s Hospital. All the research was carried out in line with the provisions of the Declaration of Helsinki of 1975.

### Cell culture and transfection

The human renal cancer cell lines 786-O, A498, 769 P, ACHN, and OSRC-2 were purchased from American Type Culture Collection (ATCC). All the cell lines were cultured in the recommended growth medium added with 10% FBS (fetal bovine serum) at 37°C with a humidified atmosphere of 5% CO_2_. The control overexpressing plasmid and ENTPD3-AS1 overexpressing plasmids were constructed by Generay Technologies (Shanghai, China). Cells were transfected with plasmids using FuGENE transfection reagent (Promega, Madison, WI). The transfection rate was displayed in Figs. S4 and S6. Nonspecific plasmids were used as negative controls.

### Total RNA extraction and real-time PCR

Total RNA of RCC tissues and cell lines was extracted by trizol reagent (Takara, Japan). One microgram total RNA was reverse transcribed by PrimeScript RT Reagent Kit (Takara, Japan). Real-time PCR was performed using ABI reagent (Thermo Fisher Scientific, West Palm Beach, FL) by StepOnePlus real-time PCR system (Applied Biosystems, Foster City, CA). 2^−ΔΔCt^ method was used to quantify the relative expression. β-actin was used as an internal control.

### Western blot

Protein was extracted by RIPA lysis buffer containing a protease inhibitor mixture (protease inhibitors; phosphatase inhibitors; PMSF; KangChen, China). The concentration of protein was quantified by BCA Protein Assay Kit (Thermo Fisher Scientific, West Palm Beach, FL). About 30 μg of protein was separated by 10% SDS-polyacrylamide gels and then transferred to PVDF membranes (Biorad, Hercules, CA). After blocked with 5% BSA for 2 h, the membranes were incubated with primary antibody rabbit anti-HIF-1α (1:1000 dilution, CST, Boston, MA) and anti-β-actin (1:20000 dilution, Sigma, Louis, MO) at 4 °C overnight. Then, the membranes were washed with TBST 5 times and incubated with species-specific secondary antibodies (1:3000 dilution, Kangcheng, China) for 1 h the next day. Secondary antibodies were labeled with HRP. The ECL detection system (Biorad, Hercules, CA) was used for visualization. Antibody against β-actin was used as an internal control.

### Chromatin immunoprecipitation

Chromatin Immunoprecipitation (ChIP) assays were conducted using the ChIP Assay Kit (Millipore, New Bedford, MA) according to the manufacturer’s protocols. 769 P and OSRC-2 cells were seeded into a 10 cm culture dish. A total of 1 × 10^7^ cells were fixed with 1% formaldehyde and quenched with 0.125 M glycine. Cells were then collected using SDS lysis buffer and sonicated to shear DNA to lengths between 200 and 800 base pairs. The DNA–protein complexes were pre-cleared with Protein A Agarose/Salmon DNA and then immunoprecipitated with anti-ZNF8 antibody (Novus Biologicals, USA), anti-Pol II antibody (Novus Biologicals, USA), and normal rabbit/mouse IgG. The co-precipitated DNAs were purified using phenol/chloroform. The extracted DNA was used for further PCR and qPCR analysis.

### Luciferase assay

The fragments were synthesized each into the cloning site of pGL3-LRF and pGL3-LRR at Generay Technologies (Shanghai, China). RCC cells were seeded in 96-well plates and transfected with 500 ng indicated plasmids and 100 ng pRL-TK plasmid (Renilla luciferase) using FuGene HD (Promega, Madison, WI). The relative firefly luciferase activity and Renilla luciferase activity were detected using the Dual-Luciferase Reporter Assay System (Promega, Madison, WI) and measured by FLUOstar Omega (BMG LABTECH, Offenburg, Germany) 24 h after transfection. The results were shown in the form of relative firefly luciferase activity normalized to Renilla luciferase activity. pGL3-control vector was used as a positive control and pGL3-LRF and pGL3-LRR were used as negative controls.

### Motif analysis

Two web tools FIMO (http://meme-suite.org/tools/fimo) and JASPAR (http://jaspar.genereg.net/) were used to analyze the effect of rs67311347 on the transcription factor binding motifs. SNP rs67311347 and its flanking sequences overlapped with ZNF8 motifs.

### Cell proliferation assay

Control and transfected RCC cells were plated into 96-well culture plates (2000 cells/well). Cell Counting Kit-8 (Dojindo, Japan) was added to the cells at specific time points (24, 48, 72, 96, and 120 h after cell plantation). After being incubated with CCK-8 reagent for 2 h away from light, the absorbance was measured by OD at 450 nm wavelength.

### Colony formation assay

Control and transfected RCC cells were seeded into 6-well culture plates (600 cells/well). After 10 days of incubation, the cells were fixed with 4% paraformaldehyde for 20 min, stained with 0.1% crystal violet for 20 min, washed with PBS 5 times, and air-dried. Finally, the colonies were counted.

### Statistical analysis

The comparisons of data between two groups were performed using Student’ *t* test, and one-way analysis of variance (ANOVA) test was used for the comparisons among three or more groups. For the clinicopathologic analysis, the Chi-square test or Fisher exact test (two-sided) was performed. The Kaplan–Meier curve and log-rank test were used to estimate overall survival and progress-free survival. All *p*-values were two-sided unless otherwise specified. All statistical analyses were performed using R-3.6.3.

## Supplementary information

The association between rs67311347 and ENTPD3-AS1 in multiple normal tissues according to the GTEx project.

The prognostic value of ZNF8 in the overall survival of TCGA-KIRC.

The prognostic value of ENTPD3-AS1 in the overall survival of TCGA-KIRC.

The transfection rate of ENTPD3-AS1 plasmid in 786-O and A498 cells.

The prognostic value of miR-155-5p in the overall survival of TCGA-KIRC.

lncRNA ENTPD3-AS1 promoted HIF-1a expression through miR-155-5p.

## Data Availability

The data that support the findings of this study are openly available in GDC at https://portal.gdc.cancer.gov/.
