# Supplementary figures and images for: SNP-mediated lncRNA-ENTPD3-AS1 upregulation suppresses renal cell carcinoma via miR-155/HIF-1α signaling
Source: Cell Death Dis. 2021 Jul 3;12(7):672. doi: 10.1038/s41419-021-03958-4 (PMC8254807; doi:10.1038/s41419-021-03958-4)

Figure S1

A

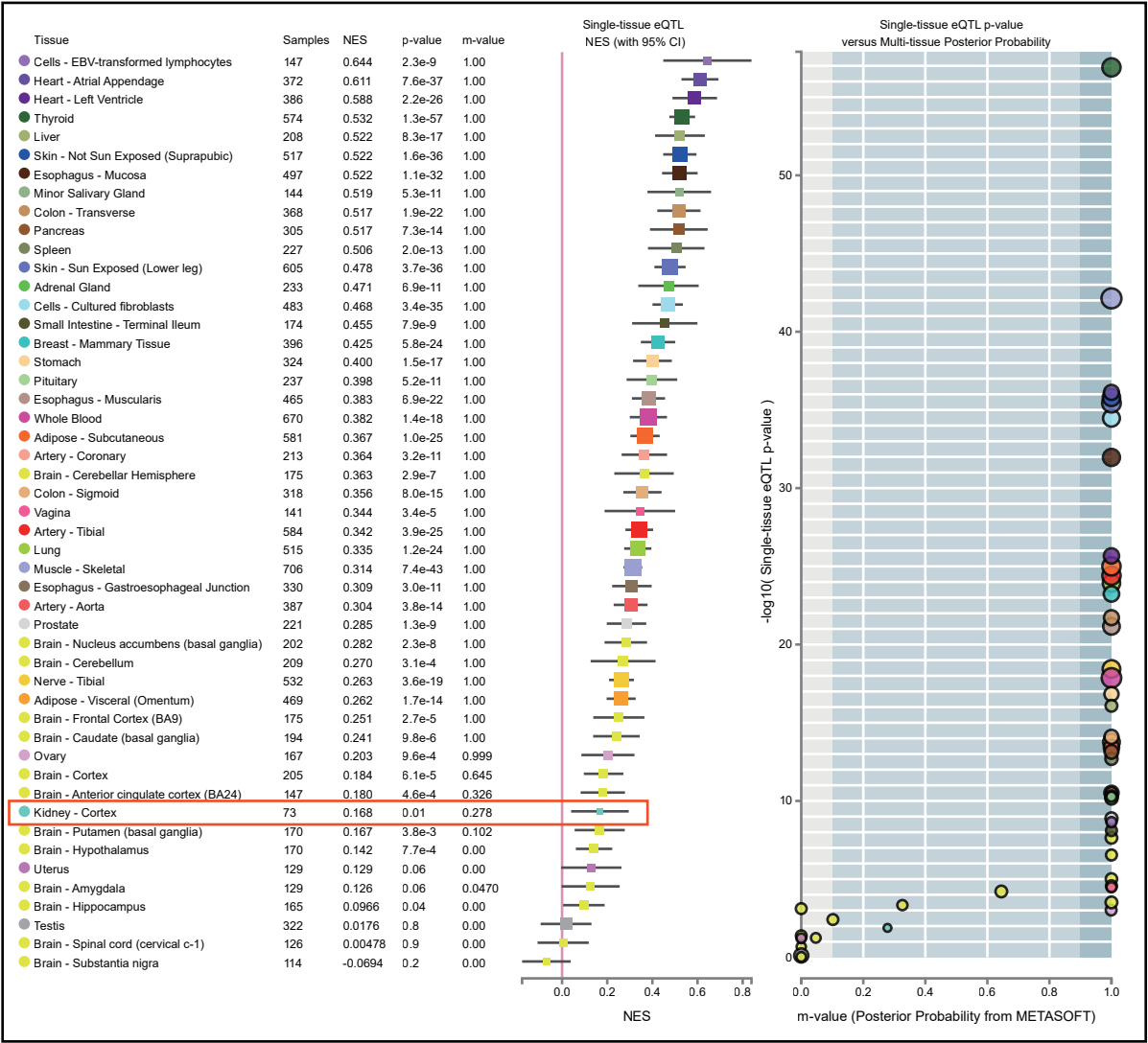

Supplement: Supplementary file 1 — The association between rs67311347 and ENTPD3-AS1 in multiple normal tissues according to the GTEx project. [file 41419_2021_3958_MOESM1_ESM.pdf]

Figure S2

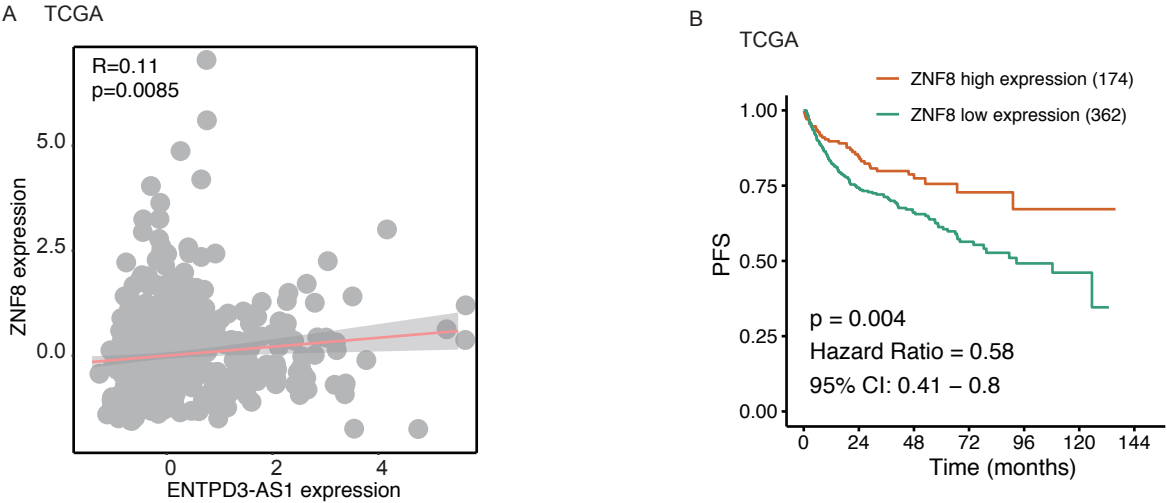

Supplement: Supplementary file 2 — The prognostic value of ZNF8 in the overall survival of TCGA-KIRC. [file 41419_2021_3958_MOESM2_ESM.pdf]

Figure S3

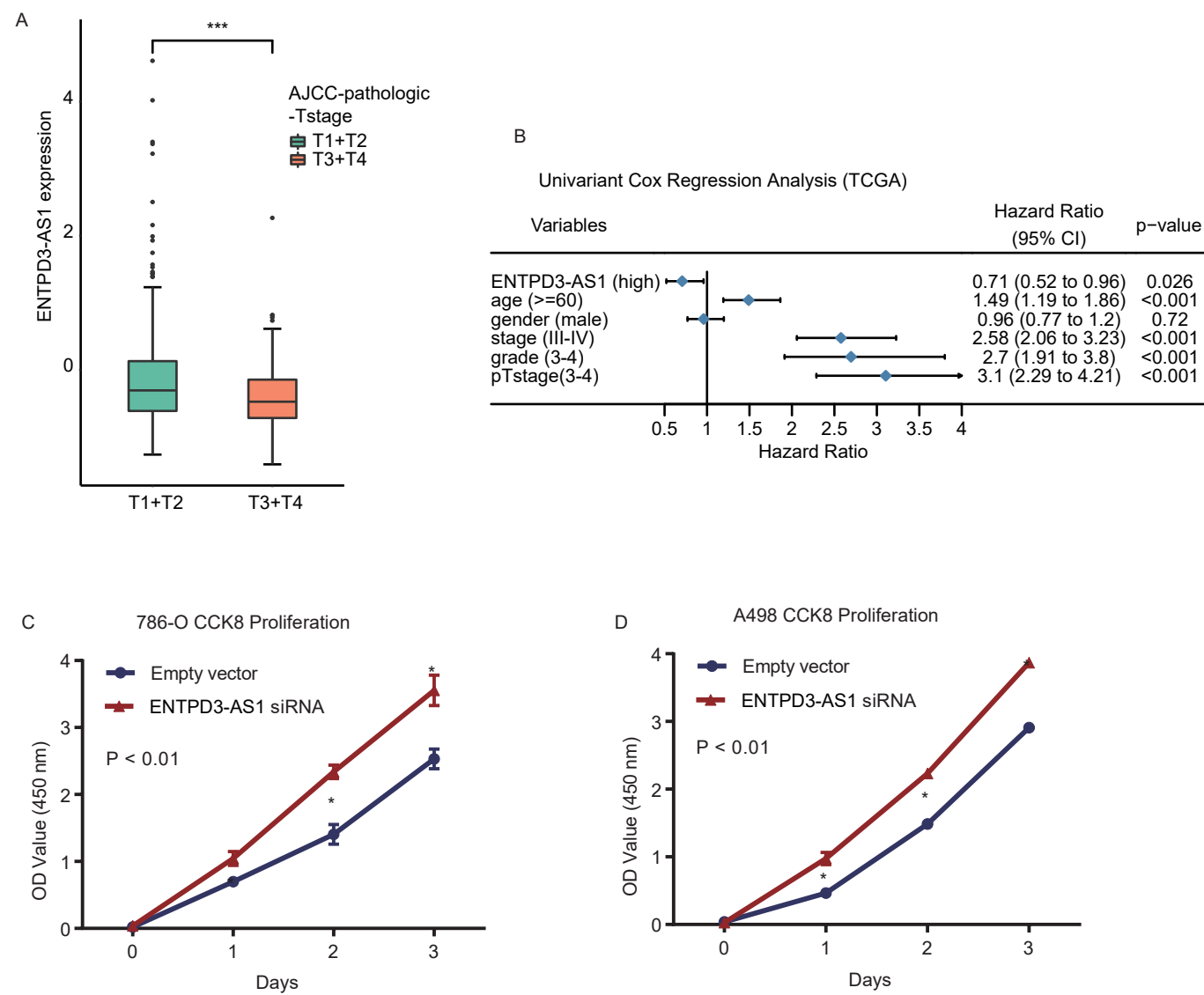

Supplement: Supplementary file 3 — The prognostic value of ENTPD3-AS1 in the overall survival of TCGA-KIRC. [file 41419_2021_3958_MOESM3_ESM.pdf]

Figure S4

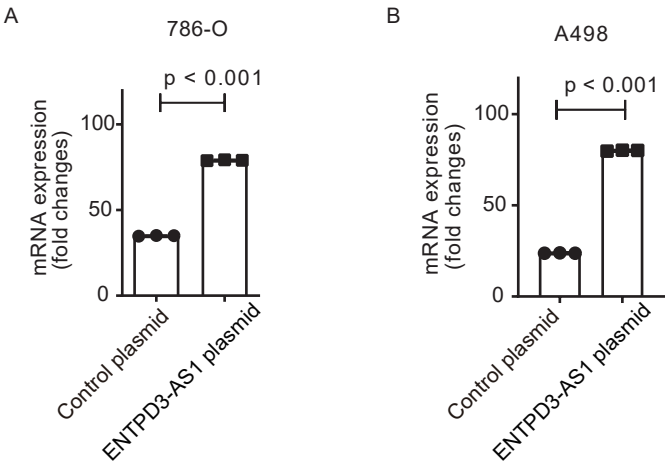

Supplement: Supplementary file 4 — The transfection rate of ENTPD3-AS1 plasmid in 786-O and A498 cells. [file 41419_2021_3958_MOESM4_ESM.pdf]

Figure S5

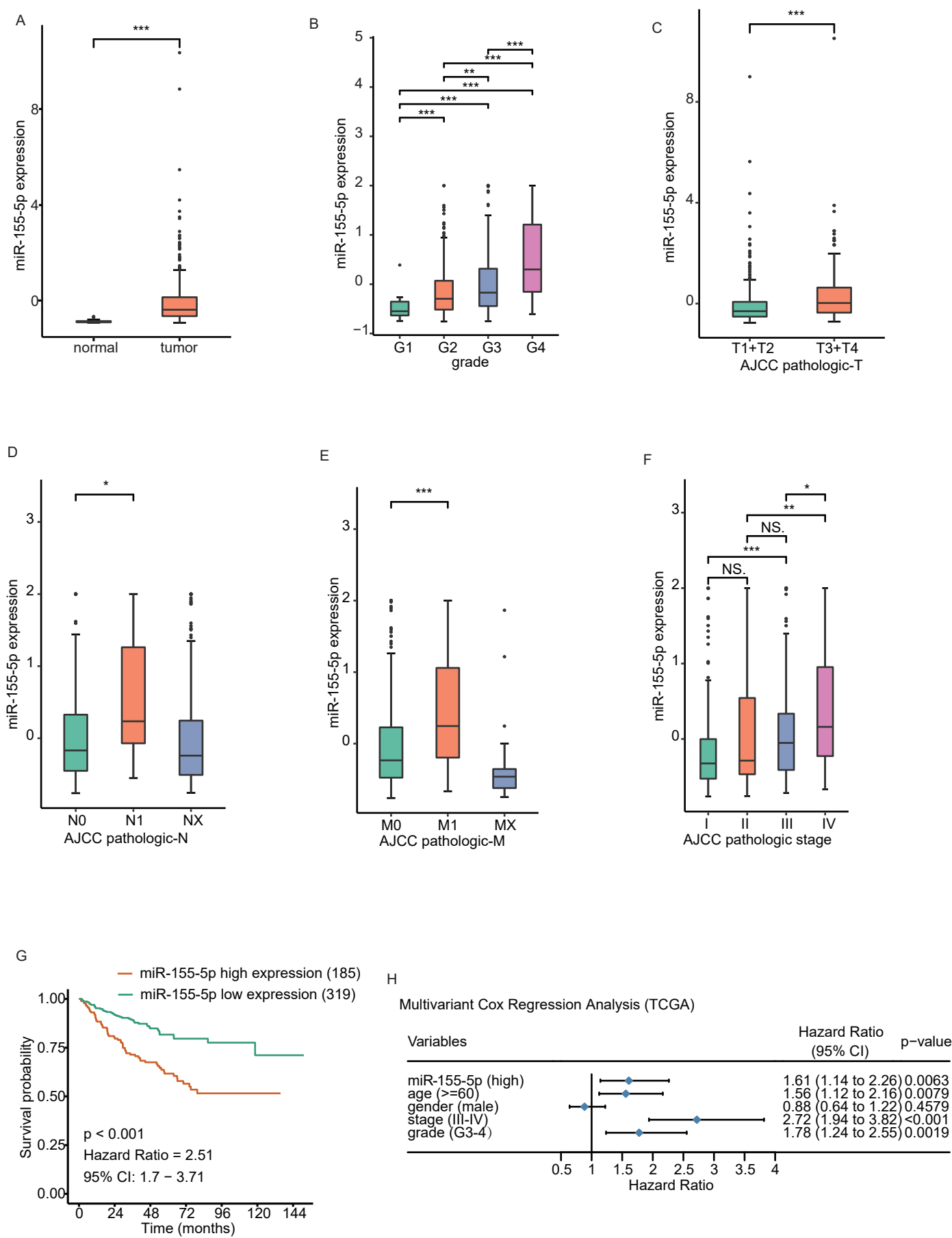

Supplement: Supplementary file 5 — The prognostic value of miR-155-5p in the overall survival of TCGA-KIRC. [file 41419_2021_3958_MOESM5_ESM.pdf]

Figure S6

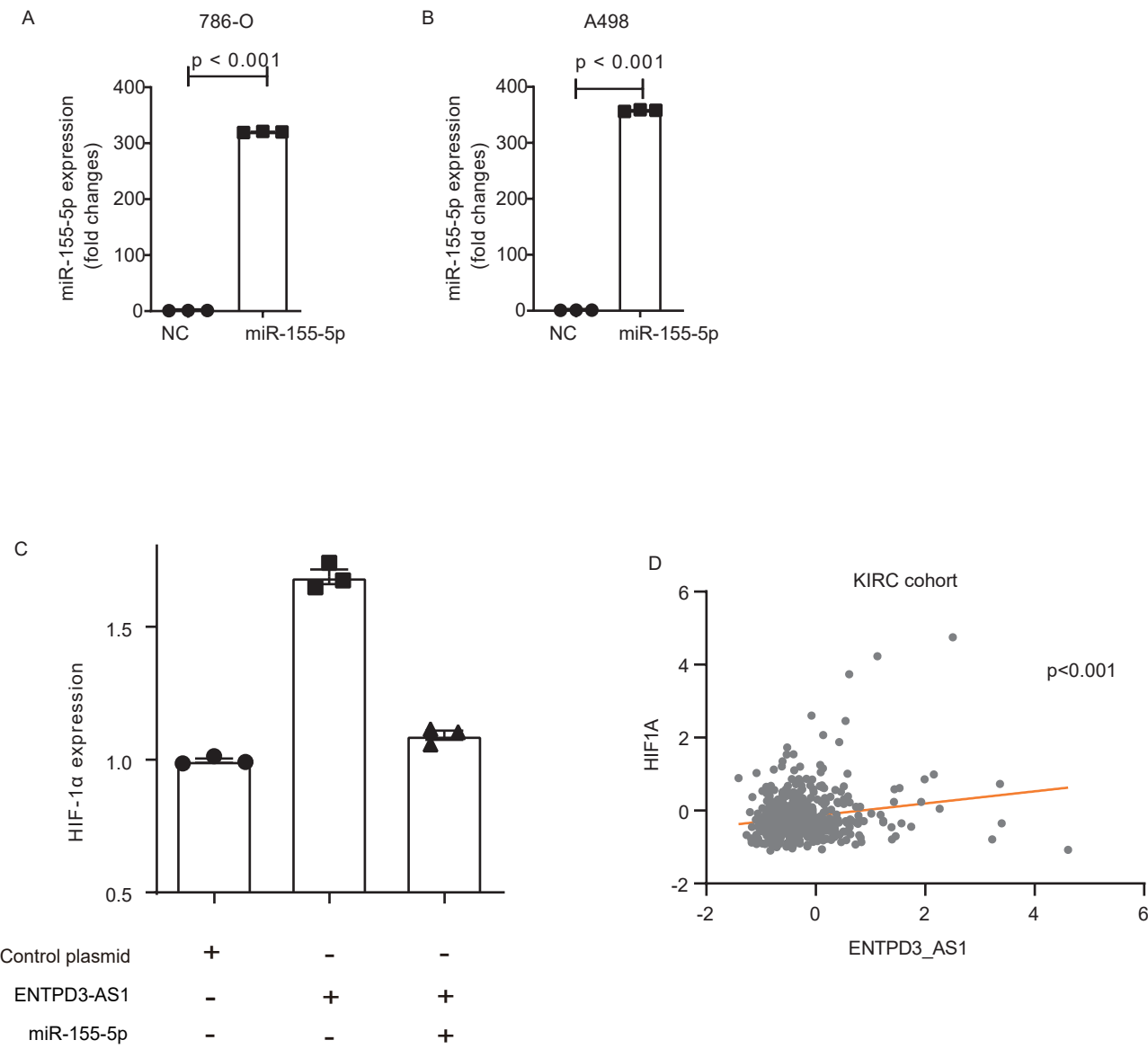

Supplement: Supplementary file 6 — lncRNA ENTPD3-AS1 promoted HIF-1a expression through miR-155-5p. [file 41419_2021_3958_MOESM6_ESM.pdf]
